# Breaking nasal epithelial cell tolerance lipopolysaccharide exposure by CD16 mediated co-stimulation with human serum immunoglobulin G

**DOI:** 10.1186/2045-7022-5-S4-P4

**Published:** 2015-06-26

**Authors:** Korneliusz Golebski, Danielle van Egmond, Esther de Groot, Jeroen den Dunnen, Wytske Fokkens, Cornelis van Drunen

**Affiliations:** 1 Academic Medical Center, University of Amsterdam, Otorhinolaryngology, Amsterdam, Netherlands; 2 Academic Medical Center, University of Amsterdam, Cell Biology, Amsterdam, Netherlands

## Background

Nasal epithelial cells are the first line of defence against invading microbes. In everyday life, we are constantly exposed to variety of bacteria and viruses, but not every exposure leads to a development of pro-inflammatory responses of nasal epithelium. Although triggering of an individual PRR is known to induce cell responses, it has become clear that the ultimate profile of cytokines production strongly depends on the cross-talk between different receptors. We have previously shown that exposure of nasal epithelium to viruses enhances its responses to Gram-positive bacteria. Since pathogenic bacteria are commonly tolerated in the human nose, we sought to characterize the nasal epithelium responses to LPS, a major component of Gram-negative bacteria cell wall.

## Methods

We exposed primary nasal epithelium isolated from 5 healthy individuals to TLR-4 agonist LPS (range:10 pg/mL to 10 μg/mL) and to human serum IgG (100 μg/mL), IgA (1 μg/mL), or IgE (1 μg/mL) in a time course over 24 hours. CD16, CD32, and CD64 receptors were blocked by preincubating the cells with 20 μg/mL of specific antibody for 30 minutes at 37°C. Expression of IL-6 and IL-8 was analysed by q-PCR and their production levels were determined by ELISA.

## Results

Despite the presence of the LPS receptor complex (TLR-4, CD14, and MD-2), 24-hour exposure of nasal epithelium to LPS did not induce IL-6 or IL-8 production at either mRNA or protein level. However, cell co-stimulation with IgG resulted in a 1.9 to 3.4 (p < 0.01) or 1.6 to 4.3 (p < 0.05) fold amplification of the IL-6 and IL-8 protein production respectively, depending on LPS concentration and inter-individual differences in responses when compared to cell responses to LPS alone. At the mRNA level, synergistic responses were even more pronounced and enhanced IL-6 expression 2.6 to 13.2 fold (p < 0.05) and IL-8 by 2.2 to 15.0 fold (p < 0.05). Importantly, IgG itself did not induce cytokine production. Cell responses to LPS were not amplified by co-stimulation with IgA or IgE. Moreover, the IgG-enhanced cell responses to LPS were abrogated to LPS-alone induction levels of IL-6 and IL-8 after blocking the CD16 (p < 0.05), but not CD32 or CD64 receptors.

## Conclusion

The data demonstrate that LPS-induced cytokine production by nasal epithelium is more complex than previously considered and show that the cross-talk of CD16 and TLR-4 may be important for the induction of Gram-negative bacteria specific immunity.

